# In Search of Mercury Lost from Sediments in a Previously Contaminated Coastal Area, Harboøre Tange, Denmark

**DOI:** 10.1007/s00128-023-03814-5

**Published:** 2023-10-08

**Authors:** Poul Bjerregaard, Christina Lisby Jensen, Anna Victoria Rode Juhl, Alexander Jacob Rahbek Markussen, Sanne Ravnholt Poulsen

**Affiliations:** 1 Department of Biology, University of Southern Denmark, Campusvej 55, Odense, DK-5230 Denmark; 2 Present Address: Cowi A/S, Vestre Stationsvej 7, Odense C, DK-5000 Denmark; 3 Present Address: Danish Environmental Protection Agency, Tolderlundsvej 5, Odense C, DK-5000 Denmark

**Keywords:** Mercury, Sediment, Biota, Benthic

## Abstract

Concentrations of mercury in sediment and benthic invertebrate fauna of Nissum Broad, North-western Jutland, Denmark were investigated. The western coast of Nissum Broad is Harboøre Tange, along which heavy mercury contamination - caused by discharge from production of mercury containing seed dressers in the 1950 and 1960s – was documented in the 1980s. Recent investigations showed marked decreases in mercury contamination in the near shore sediments along Harboøre Tange since the 1980s and the present investigation was initiated to learn if the loss of mercury from Harboøre Tange had led to an increased mercury contamination in the neighbouring marine area, Nissum Broad. Mercury concentrations in the surface sediment correlated with the content of organic matter and the slope of the regression is a good indicator for the degree of mercury contamination. Average mercury concentrations in the upper 5 cm of the sediments ranged between 0.9 and 71 ng g^− 1^ dry weight (dw) with only 1 station exceeding the Background Assessment Concentration of 70 ng g^− 1^ dw. Average mercury concentrations in blue mussels *Mytilus edulis* (169–260 ng g^− 1^ dw) and periwinkles *Littorina littorea* (66–203 ng g^− 1^ dw) exceeded those in uncontaminated areas and the Environmental Quality Standard of approximately 100 ng g^− 1^ dw. Present sediment mercury concentrations in Nissum Broad are approximately half of what they were in the 1980s, rendering it unlikely that mercury lost from Harboøre Tange has been deposited there. Sediment and organism concentrations did not show any correlation.

## Introduction

**Fig. 1:**
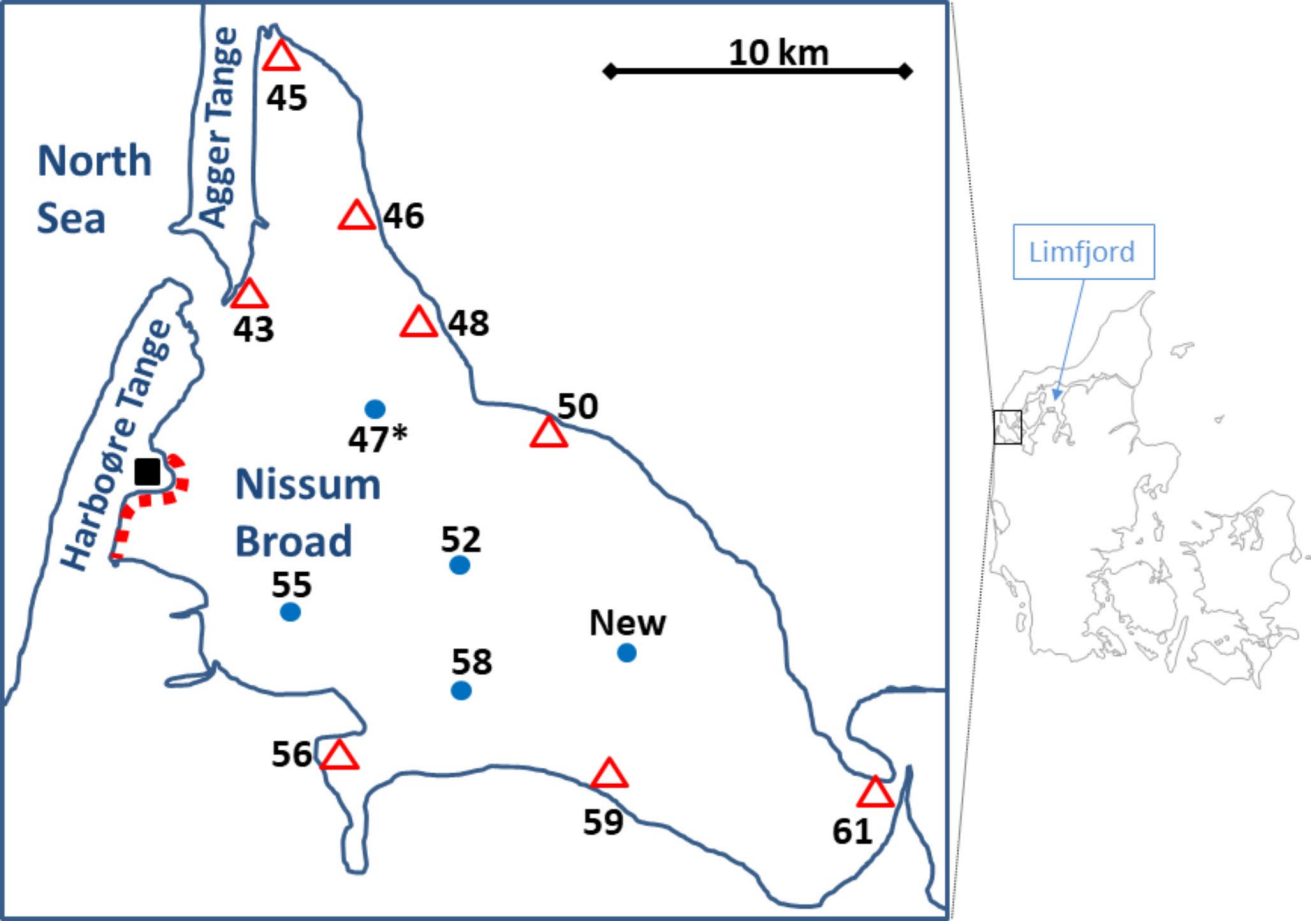
Maps showing the sampling sites in Nissum Broad. **∆**: Samples taken from the shore. ●: Samples taken at deeper waters from ship. ■: The position of the peninsula, Rønland, with the demolished chemical factory from where the contamination with mercury originated. The red dotted line indicates the area along the southeastern coast of Harboøre Tange most heavily contaminated with mercury in the 1980s

The western part of Limfjorden, Denmark, Nissum Broad along Harboøre Tange (Fig. 1) was contaminated with mercury during the 1950 and 1960s - mostly due to releases from the pesticide-producing factory, Cheminova (Lyngby and Brix 1987), situated on Rønland at Harboøre Tange. It is estimated that approximately 30 tonnes of mercury were discharged into the Nissum Broad with process waste water, lost by production mistakes and deposited in the vicinity of the factory (Kiorboe et al. 1983).

Investigations during the 1980s documented highly elevated concentrations of mercury in sediment and biota along Harboøre Tange (Andersen 1992; Brix and Lyngby 1984; Kiorboe et al. 1983; Lyngby and Brix 1987; Riisgard 1984); concentrations exceeded natural background concentrations by more than 3 orders of magnitude at the most contaminated site (Lyngby and Brix 1987). In the general Nissum Broad, mercury concentrations in blue mussels *Mytilus edulis* were also elevated compared to background levels during the 1980s (Brix and Lyngby 1984; Lyngby and Brix 1987; Riisgard and Randløv 1982) – but far from the level at the more contaminated eastern coast of Harboøre Tange.

Coastal and estuarine sediments have long been recognized as sinks for pollutants, and once buried in the sediments, metals and non-degradable, organic contaminants may persist for years or decades (e.g. Baeyens et al. 2005; Ridgway and Shimmield 2002). Therefore, it was somewhat surprising to find (Bjerregaard et al. 2020) that the concentrations of mercury in the near-shore, coastal sediments along Harboøre Tange had decreased considerably since the 1980s.

The fate of the mercury that has disappeared from the near-shore, coastal sediments along Harboøre Tange is not known. The purpose of the present study was to investigate if the disappearance of mercury from the most contaminated sites could be traced as increases in the concentrations of sediments and benthic biota in the marine area neighboring Harboøre Tange, the Nissum Broad.

## Materials and Methods

### Field Sampling

The marine area Nissum Broad (Fig. 1) constitutes the western part of the Limfjord, covering approximately 200 km^2^ with a maximum depth of 9 m. Sampling stations were situated both at shallow water in walking distance from the shore and in the deeper areas from ship (Fig. 1). Sampling locations were selected to be as close to the stations also investigated in 1982 (Brix and Lyngby 1984; Lyngby and Brix 1987). The positions of the sampling localities of the coastal sites used in 1982 by Brix and Lyngby were described in relation to fix points on the shore (and not described by precise coordinates). The locations of the stations used in the present investigation were read from the maps in Brix and Lyngby (1984), for stations in both shallow and deeper waters. The sediment samples from the deeper areas of Nissum Broad (stations 47*, 52, 55, 58 and New) were collected on March 12, 2018, from a Marine Home Guard vessel. The station named ‘New’ was not included in Brix and Lyngby’s 1982 investigation. The water depth at Brix and Lyngby’s (1984) station 47 was too shallow for the Marine Home Guard vessel to approach; therefor, station 47* was situated at some distance from the 1982 station 47. Where possible, sediment samples were collected by means of a Kajak sampler (50 cm polystyrene tube with a diameter of 5.2 cm) attached to a 5 to 8 m rod. At stations 52, 55 and New, an additional surface sample was collected by means of a van Veen grab. At station 58, the sediment was too compacted to get a sample with the Kajak sampler and only a van Veen surface sample was collected.

Samples along the coast were collected at walking depths close to the shore at water depths of 20 to 100 cm. Sediment samples at stations 46, 48, 50, 56, 59 and 61 were collected on April 5 and 6, 2018. The method of sediment sampling (Kajak samplers) was the same as used by Lyngby and Brix (1987) with which comparisons are made.

Biota was sampled at station 43, 45, 46, 48, 50,56, 59 and 61 on February 11 and 12, 2019. Specimens of the benthic fauna in an area up to 10 m from the site of the sediment sample taken at the coastal stations were collected by hand, spade, and net. If abundant, up to 15 specimens of each species were collected – otherwise 1 to 4 specimens were collected. The benthic fauna was collected as intact organisms.

### Treatment of Samples

The sediment cores were split into 1 cm fractions for the upper 5 cm and 2 cm fractions below that. The fractions were weighed, frozen, freeze dried (Labconco; Freezone) and weighed again to determine the water content. A subsample of the freeze-dried sediment was heated to 500 °C for 5 h in a Nabertherm oven to determine the organic content (Loss on Ignition – LOI). Duplicate subsamples of each freeze-dried sediment fraction were used for mercury analysis.

The biota was transported the 250 km to the laboratory in buckets with aerated water from the sampling site and processed the day after the sampling. The soft parts of the mollusks were extracted. Shrimps and other animals were weighed and treated as whole organisms. The samples were frozen at -18 °C and subsequently freeze dried (Maxi Dry Lyo).

### Mercury Analysis

Total mercury was determined by means of a Milestone DMA-80 Direct Mercury Analyser. The quality of the determinations was validated by incorporation of a certified reference material from the Canadian National Research Council (TORT-standards; lobster hepatopancreas) in each series. The TORT-standard has a certified value of 270 ± 60 ng Hg g^-1^. Sixty-nine determinations gave a mean value of 292 ± 1.5 ng Hg g^-1^ (mean ± SEM). Blanks were included in each series. Where possible, 50–100 mg freeze dried biota samples were analysed. The mercury concentration was determined in each individual specimen. Up to 700 mg dry weight sediment samples were used in the analysis. All concentrations are given on dry weight (dw) basis; sediment concentrations were also normalised regarding organic content.

### Data Handling and Statistics

Analyses of variance were used to identify differences between sampling sites; a few data sets had to be log_10_-transformed to obtain normal distributions. Tukey’s posttest was used for pairwise comparisons. The results of the statistical analyses are indicated in the figures; data points with no common lower-case letter are significantly different. Pearson correlation analyses were used to identify relations between parameters. 0.05 was used as significance level. SYSTAT© ver. 13 was used in the statistical analyses. Data were compared with the data from the investigations in the 1980s (Brix and Lyngby 1984; Lyngby and Brix 1987) but because raw data from the 1980s’ investigations were not available, it was not possible to perform a formal statistical comparison. Data from Nissum Broad were compared statistically with data from Harboøre Tange and data from areas with no known history of mercury contamination (data from Bjerregaard et al. 2020).

## Results

### Sediments

**Fig. 2:**
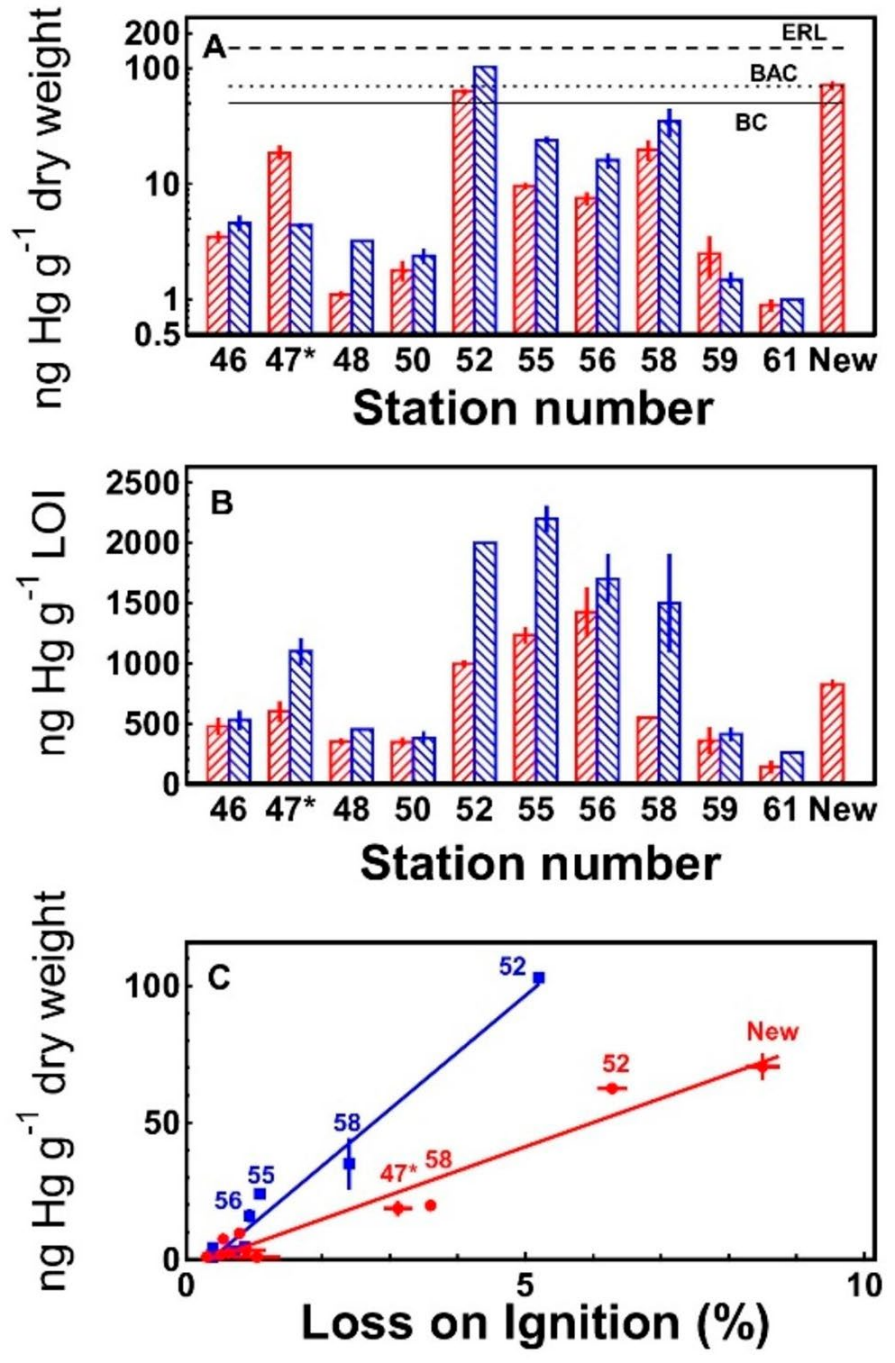
Mercury concentrations in the upper 3–5 cm of the sediment at 11 stations in Nissum Broad expressed on the basis of dry weight (**A**) and organic content (**B**). The sample at station 58 was collected with a van Veen grab with no specification of actual depth. The relation between mercury concentrations and organic content is shown in **C**. **/////** and ●: Present values. **\\\\\** and ■: 1980s values from Brix and Lyngby (1984). Sampling locations are shown in Fig. 1. BC, BAC and ERL: Background Concentration, Background Assessment Concentration and Effect Range Low defined by OSPAR (2016)

Mercury concentrations in the upper 3–5 centimeters of the sediment in Nissum Broad ranged from 0.9 to 71 ng Hg g^− 1^ dry weight (Fig. 2A). When expressed on the basis of organic content, variability in sediment mercury concentrations (138–1426 ng Hg g^− 1^ LOI) was considerably lower (Fig. 2B) and a highly significant (r = 0.97; p < 0.001) correlation ([Hg] = 8.8 *LOI – 3.0) between the organic content and the mercury concentration in the sediment of the stations was found (Fig. 2C). Data from Brix and Lyngby (1984) from Nissum Broad in the 1980s revealed a similar correlation – although with a slope approximately twice as high as in 2018 ([Hg] = 20.8 * LOI – 7.4 (r = 0.987); Fig. 2C).

**Fig. 3:**
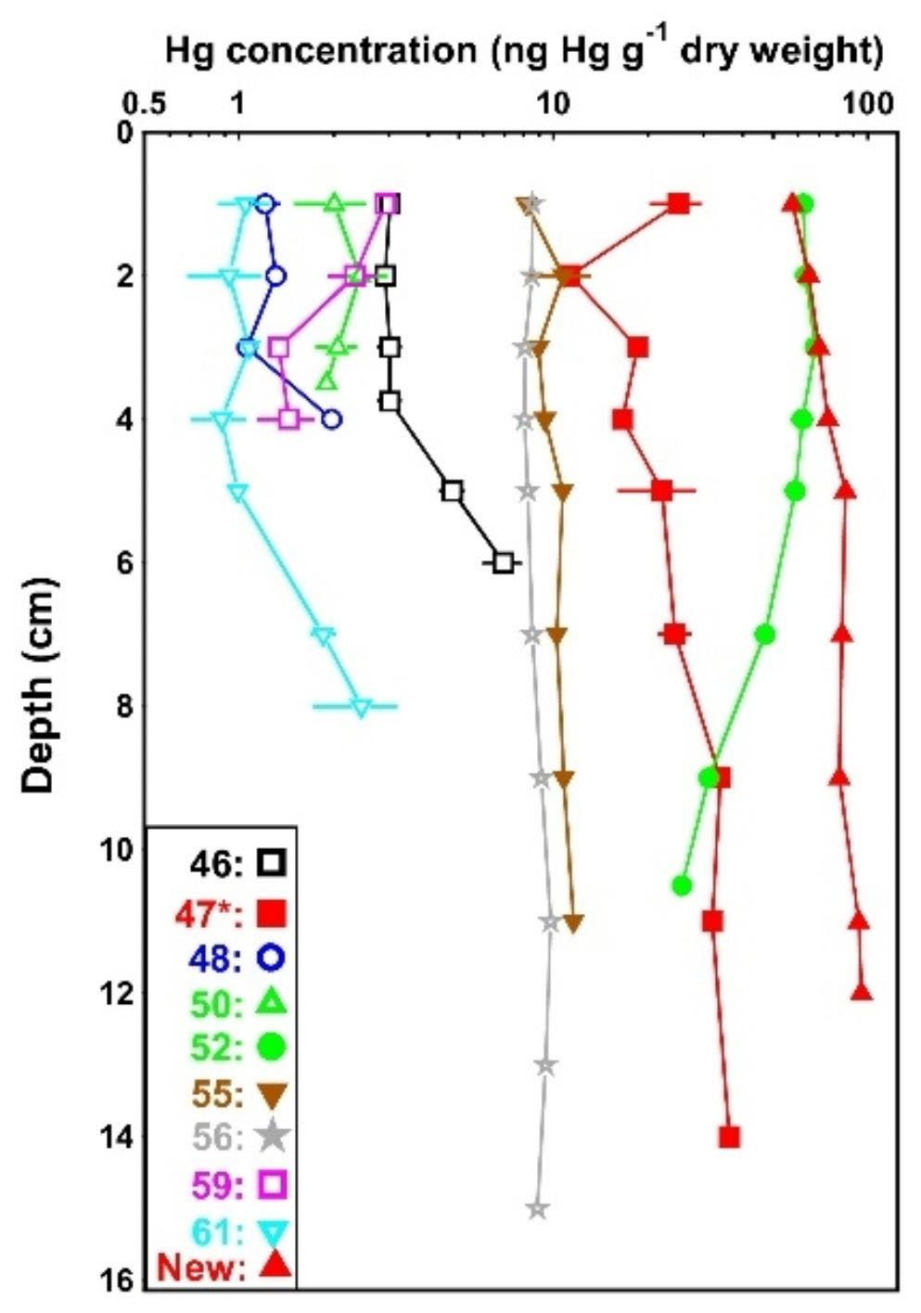
Depth profiles for mercury at the sampling sites in Nissum Broad. Means ± SEM for duplicate determinations are shown

Mercury concentrations varied significantly with depth at all stations except two (station 48 and 50), but because mercury concentration decreased with depth at some stations (52 and 59) and increased at others (46, 47, 48, 56, 61 and New), no consistent pattern was observed (Fig. 3). Some correlations between mercury concentrations and organic content were identified: the content of organic material increased significantly with depth at stations 46 and 47 (p = 0.012 and p = 0.010, respectively) and this was also reflected in a strong correlation between depth and mercury concentrations at the two stations (p = 0.002 and p = 0.009, respectively). At station 52, both organic content (p = 0.035) and mercury content (p = 0.035) decreased with depth.

At stations 52, 55 and New, the mercury concentrations in the upper 5 cm of the sediment collected with the Kajak sampler showed reasonably good accordance with the concentrations in the surface samples collected with the van Veen grab (Kajak / van Veen: Station 52: 63 ± 1.7 / 65 ± 2.1; Station 55: 9.6 ± 0.5 / 10.0 ± 0.2; Station New: 71 ± 4.6 / 63 ± 1; not shown). Because of the relatively good accordance, values from station 58 (where only samples from the van Veen grab were available) are also presented together with the Kajak samples in Fig. 2.

### Biota

Four species (blue mussels, periwinkles, gammarids and brown shrimps) were abundant in sufficient amounts at enough stations to allow meaningful comparisons.

**Fig. 4:**
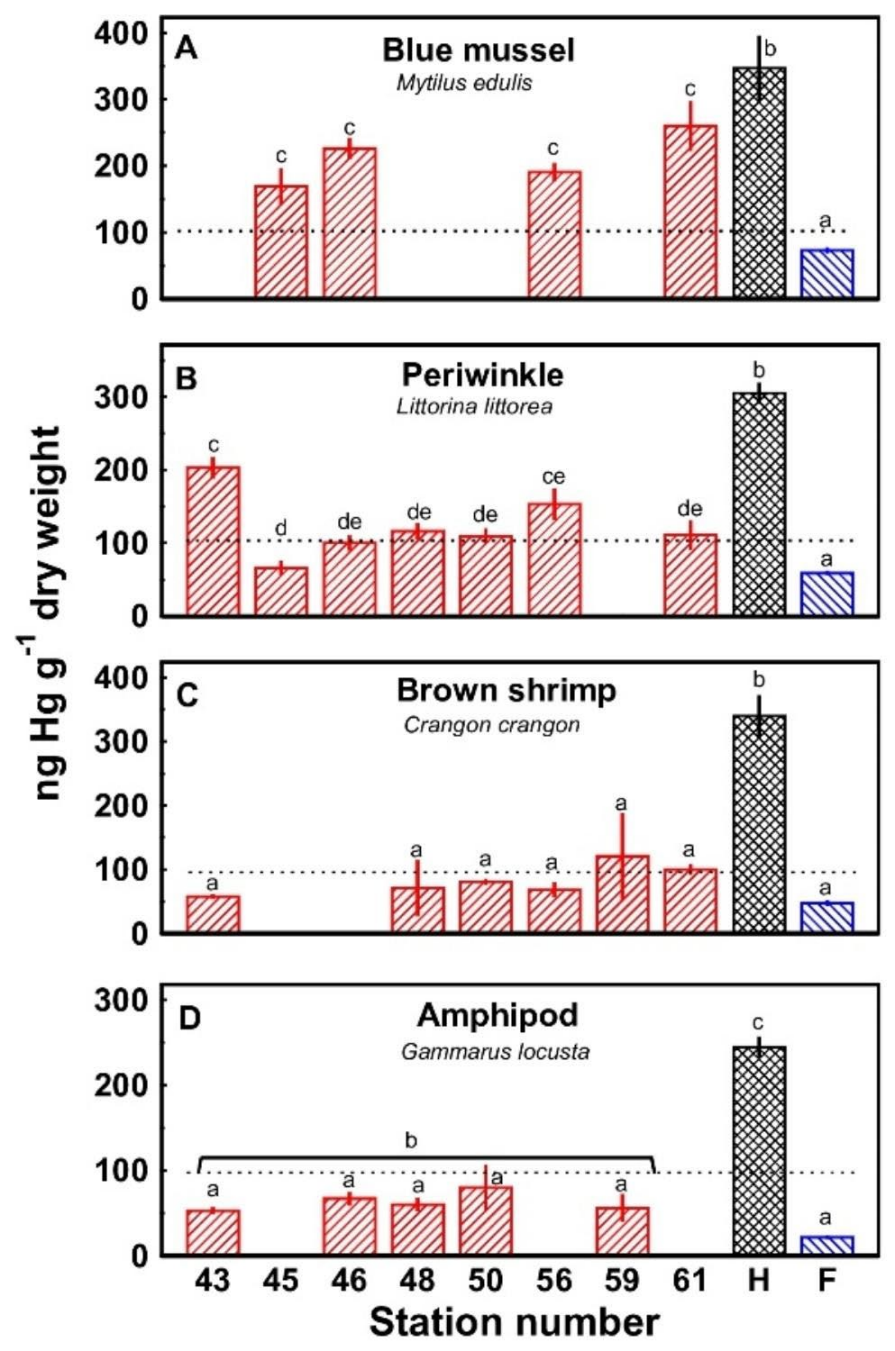
Mercury concentrations (mean ± SEM) in four species of benthic invertebrates collected at the sampling sites in Nissum Broad (**////**; n = 1–15). Data from Bjerregaard et al. (2020) for the same species at Funen reference sites (**\\\\**; n = 20–128) and the contaminated Harboøre Tange (XXX; n = 20–82) are shown for comparison. The Environmental Quality Standard is indicated by the dotted lines. Stations with no common letter are significantly different (p < 0.05)

Blue mussels were found at stations 45, 46, 56 and 61 and average mercury concentrations were 169 to 260 ng Hg g^-1^ with no statistically significant differences among the stations (Fig. 4A). Average for all the 20 mussels sampled at the 4 stations was 223 ± 13 ng Hg g^-1^. Periwinkles were found at all the coastal stations except for station 59. Average concentrations varied between 66 and 203 ng Hg g^-1^ with statistically significant differences between some of the stations (Fig. 4B). Average for all the 66 periwinkles sampled at the 7 stations was 123 ± 7 ng Hg g^-1^.

Brown shrimps were found at all the coastal stations except station 45 and 46. Average mercury concentrations for the individual stations were 58 to 121 ng Hg g^-1^ with no statistically significant differences among the stations (Fig. 4C). Average for all the 37 shrimps sampled at the 6 stations was 82 ± 6 ng Hg g^-1^.

Marine amphipods were found at stations 43, 46, 48, 50 and 59. Average mercury concentrations were 53 to 80 ng Hg g^-1^ with no statistically significant differences among the stations (Fig. 4D). Average for all the 14 amphipods sampled at the 5 stations was 63 ± 5 ng Hg g^-1^.

For mussels and periwinkles sampled at the individual Nissum Broad stations in this investigation, mercury concentrations were lower than those of Harboøre Tange and higher than those of the Funen reference sites (Fig. 4AB). Brown shrimps from the Nissum Broad had lower mercury concentrations than the shrimps from Harboøre Tange but they did not deviate from the Funen background levels (Fig. 4C). This was also true for the amphipods when the sampling stations were considered individually (Fig. 4D), but when all the 20 sampled amphipods from Nissum Broad were pooled, their mercury concentrations were different from those at Harboøre Tange (p < 0.001) and the Funen reference sites (p = 0.002).

Mercury concentrations in the biota did not appear to be related to the mercury concentrations in the upper centimetres of the sediment, neither when sediment mercury concentrations were expressed on a dry weight basis nor on an organic content basis (not shown).

## Discussion

The overall conclusion from this investigation is that mercury concentrations in the Nissum Broad generally have decreased rather than increased since the 1980s and therefor the mercury lost from the contaminated Harboøre Tange cannot be retrieved in the general Nissum Broad.

### Mercury in Sediment

It has been recognised for decades that the organic material is the most important compartment for binding of trace metals in sediments (e.g. Benoit et al. 1998; Fang and Lien 2021; Salomons and Förstner 1984) and this is also confirmed in the present investigation by the correlation between the content of mercury in the sediment samples and the organic content. Fang and Lien (2021) similarly found a significant correlation between organic content and mercury in the Danshuei River Estuary in Taiwan; the sediment of this estuary is more contaminated (80–379 ng Hg g^-1^ dw) with mercury than Nissum Broad and the slope of the regression is 115 in Danshuei versus 8.8 in Nissum. The slope of the relation between the organic content and the mercury concentration provides a good indicator of the degree of mercury contamination in a marine area – obviously together with the mercury concentration itself. It is interesting to note that the slope of the regression line expressing the relation between mercury and organic content in Nissum Broad was approximately twice as high during the early 1980s as it was in 2018, indicating that the contamination with mercury has decreased during the intervening decades. It may therefore be concluded that the loss of mercury from the near shore, coastal sediments along Harboøre Tange between the 1980s and 2014 cannot be traced as increasing concentrations in the general Nissum Broad.

Near shore, coastal sediments are known to be affected and reorganised by the effects of wave action and currents (e.g. Herman et al. 2021) and this may affect the mercury-bearing organic particles in the sediment. Mercury deposited in coastal sediments due to anthropogenic discharges has the potential to spread, as shown in several investigations: Balogh et al. (2015) showed that mercury was transported from the polluted Minamata Bay to the Yatsushiro Sea and Kudo et al. (2000) suggested that the transport was mediated by the slow (~ 110 m y^-1^) movement of mercury bearing sediment particles. In a model, storm conditions increased the mercury transport from the normal 16 kg year^-1^ to 132 kg year^-1^ (Rajar et al. 2004) – both much higher than the estimated mobilization of dissolved mercury (0.7 kg year^-1^) directly to the water phase (Akito et al. 2014).

Also, in the contaminated Gulf of Trieste model considerations and actual measurements showed that most mercury is transported adsorbed on suspended particles and that transport of dissolved total mercury is almost negligible (Rajar et al. 2004). Spread of mercury has also been observed in the Bay of Kuwait where mercury discharged to the bay during 1960s to the 1980s appears to be spreading towards the northern part of the Bay (Al-Zamel et al. 2010).

Since the loss of mercury from the near shore, coastal sediments along Harboøre Tange between the 1980s and 2014 has not led to an increase in the mercury concentration in the upper sediment layers of the general Nissum Broad, the possibility exists that the mercury lost has been transported out of the area with transport of sediment and water.

Throughout history, the Limfjord (which today actually would be named a sound) has alternated between having direct connection and no connection to the North Sea. Today, the connection to the North Sea is the app. 1 km broad Thyborøn Canal which penetrates the 1–2 km wide sandy barrier which constitutes Harboøre and Agger Tange (see Fig. 1); at the eastern side the Limfjord connects to Kattegat. The water currents in the area are mainly driven by the wind and the 30–40 cm tidal changes in the North Sea at this location. The average yearly transport of water through the Thyborøn Canal has been reported to be approximately 65 km^3^ y^-1^ westward and approximately 74 km^3^ y^-1^ eastward (resulting in a net eastward transport of approximately 9–10 km^3^ y^-1^) by the Danish Ministry of the Environment (2014). DTU-Aqua reported that the net flow in the Limfjord from west to east was 300–400 m^3^ s^-1^ (9.4–12.6 km^3^ y^-1^) but that the actual inflow and outflow from and to the North Sea is approximately 10 times higher (DTU-Aqua 2017). The volume of water in Nissum Broad is less than 1 km^3^ and with a total movement of water (east- and westward) of approximately 140 km^3^ y^-1^ this means that the water in the Nissum Broad is exchanged more than once a week.

The sampling stations along Harboøre Tange in the 2014 investigation (Bjerregaard et al. 2020) were situated in areas with shallow water affected by wave action. It is possible that wave action may bring especially the minor organic particulates with the highest mercury content (Kudo et al. 2000; Rajar et al. 2004; Yano, 2013; present investigation) into suspension whereby the water currents may have transported the mercury both to the North Sea and eastward in the Limfjord. This is probably the most plausible explanation for the decrease in the sediment concentrations along Harboøre Tange.

Mercury concentrations in the sediment of most of the sampling stations in Nissum Broad were below the 50 ng Hg g^-1^ dw defined as Background Concentration (BC) by the Oslo-Paris Convention for the Protection of the Marine Environment of the North-East Atlantic (OSPAR 2009) in 2018 and only at a few stations OSPAR’s Background Assessment Concentration (BAC) of 70 ng Hg g^-1^ dw Hg (defined by OSPAR as ‘near background’ (OSPAR 2016) were approached or slightly exceeded. Except for one sampling station (station 52) this was also true in the 1982-sampling (Brix and Lyngby 1984; Lyngby and Brix 1987). Mercury concentrations at all stations – both in 1982 and 2018 – were below the level ‘Effect Range Low – ERL’ under which OSPAR (2016) reckons there is a low risk of adverse effects in the benthic and sediment dwelling organisms. Also, concentrations were below Canadian Interim Marine Sediment Quality Guidelines (130 ng Hg g^-1^ dw) and far below their Probable Effect Levels (700 ng Hg g^-1^ dw)(CCME 1999).

### Mercury in Benthic Organisms

In their sampling in the entire Limfjord in 1982, Brix and Lyngby defined a background value for mercury concentrations in blue mussel soft parts of 108 ng Hg g^− 1^ dw and concluded that the mussels sampled in Nissum Broad in 1982 had elevated concentrations with average concentrations of 165 ± 18 ng Hg g^− 1^ dw. Likewise, blue mussels sampled in Nissum Broad – but not in the direct vicinity of the polluted Harboøre Tange − 1980–1981 showed elevated concentrations (17–26 ng Hg g^− 1^ ww) compared to mussels (4–10 ng Hg g^− 1^ ww) collected more easterly in the Limfjord (Riisgard and Randløv 1982). The average for the 20 mussels sampled from 4 stations in the Nissum Broad in the present investigation is 223 ± 13 ng Hg g^− 1^ dw, closer to the range of the 1982 values for Nissum Broad than the background values in both the general Limfjord in 1982 (Brix and Lyngby 1984; Lyngby and Brix 1987) and in the Funen reference area (73 ± 3 ng Hg g^− 1^ dw; n = 128) in 2016 (Bjerregaard et al. 2020). These data indicate that in the Nissum Broad blue mussels, mercury concentrations have not decreased since the 1980s and that they are still higher than general background values. No 1980s data on mercury concentrations in periwinkles, brown shrimps and amphipods in the general Nissum Broad are available but like for the blue mussels, present concentrations are elevated relative to general background concentrations but lower than the concentrations along Harboøre Tange. Average mercury concentrations in blue mussels *M. edulis* and periwinkles *L. littorea* (223 ± 13 and 123 ± 7 ng g^− 1^ dw, respectively) exceed the Environmental Quality Standard of ≈ 100 ng g^− 1^ dw defined by the European Union’s Water Framework Directive.

Mercury concentrations in sediment and biota do not show correlations, and the decrease in mercury concentrations in the sediment in Nissum Broad between the 1980s and 2018 is not reflected in a similar decrease in the mercury concentrations in the benthic, invertebrate biota, which still hold mercury concentrations greater than organism concentrations at reference sites. This reflects the previous observation (Bjerregaard et al. 2020) that a simple relationship between concentrations in sediment and biota does not always exist.

## Data Availability

Data sets are available upon contact to PB.
